# Food & Nutrition: What Everyone Needs to Know

**DOI:** 10.5195/jmla.2019.697

**Published:** 2019-07-01

**Authors:** Eleanor Shanklin Truex

**Affiliations:** Clinical Librarian, Medical Library, AMITA Health Saint Joseph Hospital, Chicago, IL, and AMITA Health Saint Francis Hospital, Evanston, IL, etruex62@gmail.com

P. K. Newby’s treatment of this perennial topic is somewhat daunting in its scope. As she says in her preface:

no individual book could ever do justice to this vast subject, covering in depth the myriad ways food shapes and impacts our health, environment, economy and society. I try, even so, to provide what everyone needs to know,…large bodies of literature across scientific disciplines like nutrition, agriculture, biology, and anthropology—to better understand how and why what we eat matters, from farm to fork. (p. xv)

She reiterates this at the start of the first section as well: “it is not possible for one book alone to provide ‘everything you need to know’” (p. 8), which then raises the question for using such a title.

This book is marketed to appeal to the casual lay reader, with an almost overwhelming focus on factual data. Given the chicanery present in much of nutrition and diet publishing, that is refreshing.

The book is divided into five parts: 1. “Why What We Eat Matters: From Farm to Fork”; 2. “Dining Throughout Human History: Science, Technology, Eater, Environment”; 3. “Essential Food and Nutrition: Separating Science from Junk Science”; 4. “Food, Glorious Food”; and 5. “Diets for Optimal Health, Longevity, Sustainability: Today and Tomorrow.”

Any one of these topics could be a book in itself. It is a vast scope for 293 pages. Because Newby wants to cover so much ground, her approach must be broad. In her quest to span both global and individual food issues, some things, such as the federal government bodies and policies that affect so much of the business of food, are overlooked. The lack of treatment of such federal programs as MyPlate or Dietary Reference Intakes (DRIs) is inexplicable for a book designed for lay people, especially since statistics from the US Department of Agriculture (USDA), Food and Drug Administration (FDA), National Institutes of Health (NIH), and Environmental Protection Agency (EPA) are heavily quoted. The entire industry of commercial marketing of food and food choices to people is not explored.

Each of the above-named sections contains approximately twenty-five questions that are posed and answered. Sample questions are “Is sugar addictive?;” “What’s in a nut?”; and “What is a concentrated animal feeding operation (CAFO) and why does it matter?” The topics are interesting, but again, each is worthy of in-depth treatment but does not receive it. For example, CAFOs are defined by the EPA (p. 170), but the USDA program that encourages CAFO managers to practice environmentally responsible nutrition management is not mentioned. Another example is the consumption of fish caught locally. Newby advises against eating this more than once per week due to possible (unnamed) contaminants in these fish (p. 191); specific species or differences in freshwater or saltwater fish are not delineated. The reader is left scrolling through Chapter 13’s citations in the portable document format (PDF) file, wondering which references apply to this edict.

I reviewed the separate PDF of citations that are available only on Newby’s website via a submenu. She quotes many studies on world nutrition, foodborne illness, and global agribusiness practices from medical journals, newspapers, government reports, and the *Encyclopaedia Britannica*. Her sources are varied, current, and within accepted medical literature conventions but are not tethered to her actual writings regarding the 134 questions. Why this severing of information from original sources? At the back of the book, there is a short list of selected references for each chapter, unannotated. There is also a footnoted “Notes” section on each chapter, as well as an index, so why not the complete bibliography? The “over 1200 references” are available at www.pknewby.com, something easily overlooked by any reader who does not bother with reading prefaces or introductions.

Given the broad scope of this work, it is a challenge to find a book with which to compare it. Walter Willet’s *Eat, Drink and Be Healthy: The Harvard Medical School Guide to Healthy Eating* (2017), a Harvard Medical School book codeveloped with the Harvard T.H. Chan School of Public Health (published by Free Press), seems a reasonable choice. It has a slightly different focus: it concerns itself with the pitfalls of dietary choices and the need to better understand nutrition labels; the USDA’s DRIs, the RDAs of old; and MyPlate. It is not as comprehensive a book as Newby’s, but it shares the same basic philosophy as hers and it covers the politics of food as well. It is more academic in that its bibliography is included in the book, but it is also not for clinical use.

I laud Professor Newby for her effort in producing a nutrition book that is accessible for a casual reader, but I cannot condone the severing of sources of information; it undermines the credibility of the writing. If this is a new industry custom, I hope it is short-lived. What happens to this book if her website ceases to exist? This structuring of the work does Newby a grave disservice, but the true harm is to reliable, authoritative, responsible secondary research such as hers. I cannot recommend this book to a medical or hospital library. A public library may want to have it on their shelves but will need to add a prominent sticker or notice to patrons that the references are separate from this book and to provide the website’s uniform resource locator (URL) for those who want citations.

